# Treatment of an Adult Skeletal Class III Patient with Surgically Assisted Rapid Palatal Expansion and Facemask

**DOI:** 10.1155/2019/8251903

**Published:** 2019-12-30

**Authors:** Hossein Behnia, Hossein Mohammad-Rahimi, Mohammad Behnaz

**Affiliations:** ^1^Department of Oral and Maxillofacial Surgery, Dental School, Shahid Beheshti University of Medical Sciences, Tehran, Iran; ^2^Dental Research Center, Dental Research Institute, Dental School, Shahid Beheshti University of Medical Sciences, Tehran, Iran; ^3^Department of Orthodontics, Dental School, Shahid Beheshti University of Medical Sciences, Tehran, Iran

## Abstract

This case report presents the treatment of a 21-year-old male patient with class III skeletal malocclusion, an open bite, and vertical growth pattern. He was managed with surgically assisted rapid palatal expansion (SARPE) along with an orthopedic facemask. The duration of treatment was 16 months. Significant improvement and favourable outcome were observed concerning both facial appearance and paraclinical parameters after completion of treatment.

## 1. Introduction

Excessive length of the mandibular body, maxillary hypoplasia, or a combination of both can lead to skeletal class III malocclusion [1, 2]. It has a reported incidence of 5% to 14% in different populations [3]. The treatment of skeletal class III malocclusion in adults is challenging for orthodontists [4]. Maxillary transverse deficiency is common in these patients, which makes their management even more difficult [5].

Various treatment options have been suggested for class III patients including dentofacial orthopedics (e.g., facemask), camouflage treatment, and orthognathic surgery [6]. However, the three-step surgical-orthodontic approach has been mentioned as the gold standard for most cases of skeletal class III malocclusion, particularly adults. This technique includes a presurgical-orthodontic phase for levelling and alignment of teeth followed by an orthognathic surgery and a postsurgical-orthodontic phase to adjust the occlusion [7, 8]. Although orthodontists conventionally and widely use this approach, it has some disadvantages: it prolongs the treatment time, orthodontic decompensation may worsen the facial profile, and it is associated with patient discomfort during the presurgical-orthodontic phase due to unideal changes in occlusion [9].

Due to the reasons above, researchers have suggested alternative treatment modalities such as the surgery-first approach [9] and application of orthopedic facemask with rapid palatal expansion (RPE) [10, 11]. In recent years, the combined use of facemask and RPE has shown favourable outcomes in class III patients especially in those with a maxillary constriction [12]. However, such studies often used nonsurgical approaches for RPE. Therefore, the outcome of this technique may not be satisfactory for adult patients [13].

We hypothesized that surgically assisted rapid palatal expansion (SARPE) along with an orthopedic facemask might lead to desirable outcomes in adult patients with skeletal class III malocclusion and maxillary constriction. This case report describes the management of an adult patient with class III skeletal malocclusion, an open bite, and vertical growth pattern.

## 2. Case Presentation

### 2.1. Diagnosis and Etiology

A 21-year-old male was referred to the Orthodontics Department of the School of Dentistry, Shahid Beheshti University of Medical Sciences, in 2016. His chief complaint was dental crowding in the maxilla and unfavourable profile view. The patient mentioned that he preferred a nonsurgical treatment approach.

**Figure 1:**
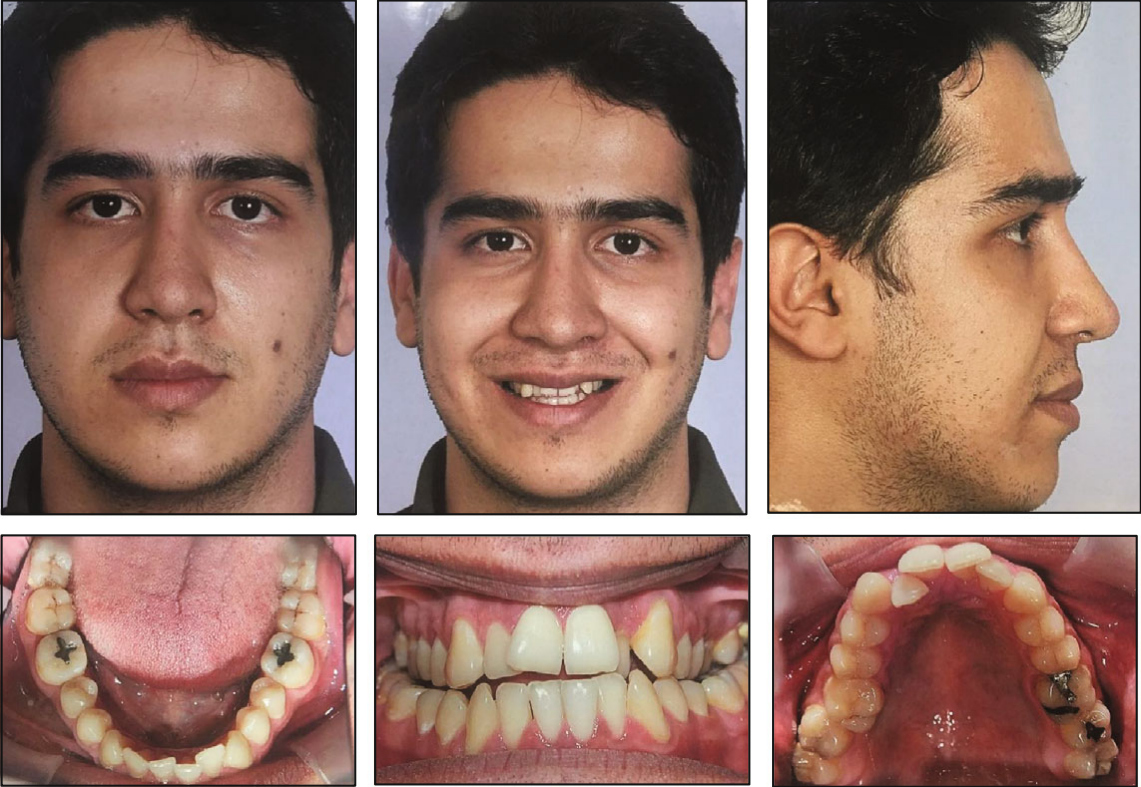
Pre-treatment facial and intraoral photographs.

Clinical examination was carried out, and a lateral cephalogram was obtained for a definite diagnosis. Moreover, a diagnostic cast was fabricated. Preoperative photography is presented in Figure 1.

**Figure 2:**
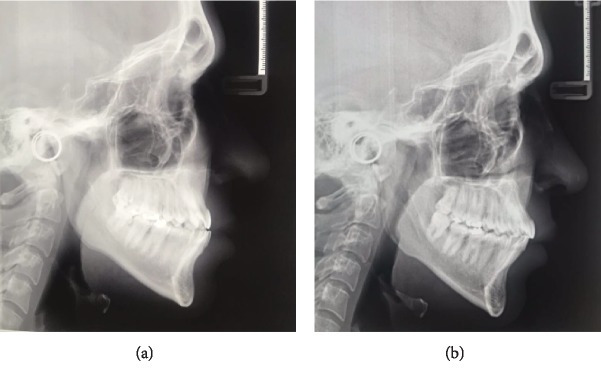
Lateral cephalometry: (a) pretreatment; (b) posttreatment.

The lateral cephalometric measurements indicated skeletal class III malocclusion pattern (ANB: -5.0°, Wits appraisal: 7.2 mm, SNA: 77.7°, SNB: 78.3°) with a vertical growth pattern (SN-MP: 42.4° and S-Go/N-Me: 57.7%). The inclination of the maxillary incisors was close to normal range (U1-SN: 99.3°); however, the mandibular incisors were retroclined because of dentoalveolar compensation (IMPA: 85.9°). The patient had a slightly obtuse nasolabial angle (103.2°), and his lower lip was protrusive relative to the E line (0.4 mm) (Figure 2(a)).

Based on clinical and radiographic examinations, the patient was diagnosed with class III skeletal malocclusion with an open bite tendency and edge-to-edge incisor relationship. The patient had maxillary constriction, which led to posterior crossbite. Dental crowding of the maxilla was also observed. Moreover, the patient had a concave profile.

The etiology of his condition was found to be hereditary. Tooth size-arch length discrepancy was also present due to maxillary constriction.

### 2.2. Treatment Objectives

Based on the diagnosis and etiologies, the treatment objectives were set as follows: (i) skeletal expansion of the maxilla, (ii) forward movement of the maxilla, (iii) correction of posterior crossbite, (iv) protrusion of the maxillary anterior teeth, (v) correction of dental crowding in the maxilla, (vi) establishing Angle's class I occlusion, (vii) correction of overjet and overbite, (viii) achieving a stable occlusal relationship, and (ix) achieving satisfying facial esthetics.

### 2.3. Treatment Alternatives

Orthognathic surgery (Le Fort I) and anterior repositioning of the maxilla is the conventional treatment for adult patients with skeletal class III malocclusion due to maxillary retrognathism. In patients with skeletal maxillary constriction, SARPE is also required in addition to Le Fort I surgery; thus, these patients need a two-step surgical procedure, which is more complex and may be associated with more complications.

Facemask has been suggested as an alternative approach to decrease the number of surgical procedures required. However, application of this approach often fails in achieving the desired outcome.

### 2.4. Treatment Progress

Due to the patient's preference, a nonsurgical approach for camouflage therapy was first started. We started RPE using a hyrax device. After four months, we decided to stop the treatment following the observation of flaring of the posterior teeth and gingival recession.

Considering the skeletal constriction of the maxilla, SARPE was considered for the patient. Initially, we waited for two months for the previous treatment outcomes to relapse. Next, full banding and bonding of the maxillary teeth were performed to correct the alignment of the maxillary teeth. Also, space was created between the maxillary central incisors, especially in the root area. This space helped to protect and preserve the roots of the incisor teeth during the surgical incision in the midpalatal region for SARPE. To create this space, a spring was placed between the central incisors. This treatment was started nine months before the surgical procedure.

After achieving the desired outcomes by fixed orthodontic treatment, SARPE was performed. Briefly, following the induction of general anesthesia, an osteotomy was done in the midpalatal region. Then, the intermaxillary suture was cut. Downfracture of the maxilla was not performed during the surgery.

Following the surgical procedure, a previously fabricated hyrax device was used for palatal expansion as a tooth-borne appliance. The active expansion continued for 12 days (twice a day). After this period, 6 mm space was achieved between the maxillary central incisors, which was desirable. The device remained in the mouth for the retention period of about four months.

A facemask was used to treat the maxillary deficiency in the sagittal plane. The facemask was connected to the hooks embedded in the hyrax device using elastics. The duration of the active phase of treatment by the facemask was three months.

Finally, occlusal settling and final detailing of dentition were performed by fixed orthodontics and intermaxillary elastics. Orthodontic treatment was accomplished about one year after surgery.

### 2.5. Treatment Results

**Figure 3:**
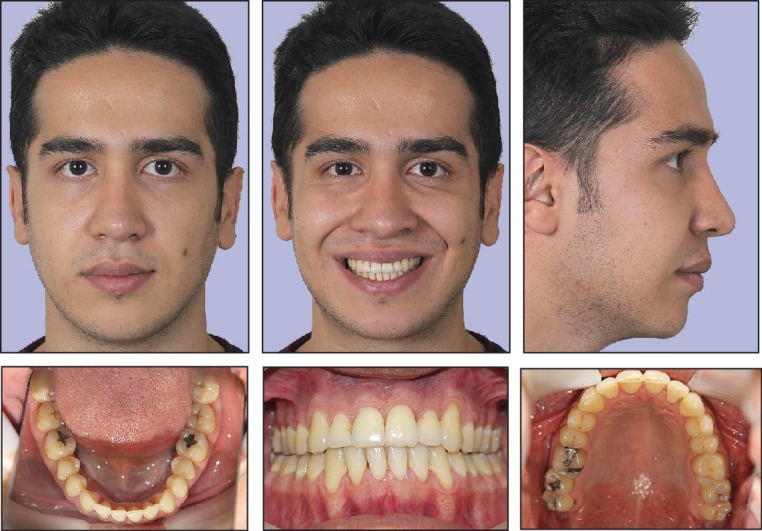
Posttreatment facial and intraoral photographs.

The treatment outcome was desirable. Skeletal relationships were appropriate. An ideal occlusal relationship was achieved. Dental crowding and posterior crossbite were corrected. No gingival recession was observed after treatment. Postoperative photography is presented in Figure 3.

Lateral cephalometric analysis showed skeletal improvement (ANB: 2.0°, Wits appraisal: 0.6 mm, SNA: 79.7°, SNB: 77.3°). SN-MP decreased from 42.4° to 39.0°, which led to less hyperdivergent growth pattern when compared to the pretreatment facial status. Moreover, as a result of treatment, the maxillary incisors were proclined (U1-SN: 107.6°) to make room for the posterior teeth. Also, the mandibular incisors were uprighted from IMPA 85.9° to IMPA 90.3° (Figure 2(b)). Detailed information of pre- and posttreatment lateral cephalometric analysis is available in Table 1.

## 3. Discussion

The management of adult patients with maxillary deficiency in addition to class III skeletal malocclusion is complicated [5]. The aim of this study was to propose a new approach for the treatment of such patients in case of failure of camouflage therapy without performing the two-step surgical procedure. The present case report showed successful management of a 21-year-old patient with class III skeletal malocclusion using a combination of SARPE and facemask, which led to a desirable outcome.

The treatment of choice for management of adult patients with skeletal class III malocclusion may include camouflage orthodontic treatment or orthognathic surgery. Camouflage therapy is the treatment of choice for nongrowing adult patients with mild or moderate class III skeletal malocclusion and favourable facial status. However, if patients have severe class III skeletal malocclusion, orthognathic surgery may be preferred [14, 15]. A favourable outcome can be expected by accurate diagnosis, proper case selection, and efficient treatment planning.

In the present case report, we first initiated the camouflage therapy. However, following the occurrence of flaring of the posterior teeth and gingival recession, we decided to change the treatment plan. According to the literature, in case of deterioration of periodontal status as a result of camouflage orthodontic treatment, the orthognathic surgical approach should be preferably adopted [16]. The occurrence of gingival recession in camouflage therapy is believed to be related to the patient's periodontal biotype. Moreover, the movement of mandibular incisors is limited in patients with thin alveolar bone due to the risk of dehiscence [17].

As mentioned earlier, the conventional orthognathic surgery for skeletal class III malocclusion patients is composed of a three-step surgical-orthodontic approach including preoperative orthodontics, orthognathic surgery, and postoperative orthodontic treatment [7]. Various problems have been reported for this approach. The long duration of orthodontic treatment especially in the presurgical phase may discourage the patients and negatively affect their compliance [18]. Dowling et al. reported that the length of conventional surgical-orthodontic treatment is about 22 months and includes 16 months of presurgical-orthodontic treatment and six months of postsurgical-orthodontic treatment [19]. However, these time periods may vary depending on the operator's skills [20]. Some other disadvantages have also been reported for this approach including the gingival recession, root resorption, and worsened facial esthetics, which may occur during the presurgical-orthodontic phase [18, 21]. To prevent them, researchers have suggested a two-step orthognathic surgical approach that does not require preoperative orthodontics [18, 22–24]. This new approach has a significantly shorter treatment period [22], which in addition to immediate improvement of facial esthetics [25, 26] often results in higher patient satisfaction and their improved cooperation.

Despite these advantages, we did not perform orthognathic surgery. In orthognathic surgery of patients with skeletal class III malocclusion, researchers recommend the bimaxillary osteotomy procedures, which include Le Fort I and bilateral sagittal split osteotomy [27]. The orthognathic surgery itself has various complications such as paresthesia, hematoma, and infection. Furthermore, only a few patients are willing to undergo orthognathic surgery [28].

We selected a three-step approach for our adult patient, which included an initial orthodontic treatment, SARPE, and facemask therapy. This approach yielded favourable results regarding facial esthetics, occlusal relationships, and cephalometric measurements. The maxillary expansion procedures can help in achieving better outcomes in the treatment of patients with skeletal class III malocclusion. Furthermore, they increase the final stability of orthodontic treatment by providing appropriate overjet for the buccal segments [29, 30].

After SARPE, we used a facemask for protraction of the maxillary segment by applying elastic forces to the hyrax. Similar to our study, it has been reported that maxillary expansion besides the application of facemask will lead to favourable outcomes in class III patients. This approach has both skeletal and dental effects on the patient's occlusion [6, 16, 31, 32].

Unlike the aforementioned studies that adopted a nonsurgical approach for maxillary expansion, we used SARPE for our patient. This surgical approach is the recommended procedure for adult patients with maxillary constriction, who have passed their growth spurt [33, 34]. Timms and Vero [35] suggested that the best time for SARPE is between 25 and 30 years of age. However, it should be noted that skeletal maturation is more important than the chronological age of the patient.

The results of the present study showed that in an adult patient with skeletal class III malocclusion and maxillary constriction, successful treatment outcomes can be achieved by a combination of SARPE and application of facemask. However, clinical trials are required to assess the efficacy of this treatment approach further.

## Figures and Tables

**Table 1:** Cephalometric measurements.

	Pretreatment	Posttreatment	Norm ± SD
Skeletal (vertical analysis)			
FH-SN (°)	6.4	4.0	6.0 ± 4.0
MP-SN (°)	42.4	39.0	33.0 ± 6.0
Sum of angles (Jarabak) (°)	402.4	399.0	380.0 ± 6.0
S-Go/N-Me (%)	57.7	624	65.0 ± 4.0
Skeletal (sagittal analysis)			
SNA (°)	77.7	79.3	82.0 ± 3.5
SNB (°)	78.3	77.3	80.9 ± 3.4
ANB (°)	-0.6	2.0	1.6 ± 1.5
Wits appraisal (°)	-5.0	0.6	−1.0 ± 1.0
Dental			
U1-SN (°)	99.3	107.6	103.1 ± 5.5
IMPA (°)	85.9	90.3	95.0 ± 7.0
Interincisal angle (U1-L1) (°)	132.4	123.0	130.0 ± 6.0
U1-NA (mm)	5.8	7.4	4.3 ± 2.7
L1-NB (mm)	5.8	7.9	4.0 ± 1.8
Soft tissue			
Upper lip to E-plane (mm)	-4.4	-2.3	−8.0 ± 2.0
Lower lip to E-plane (mm)	0.4	-0.1	−2.0 ± 2.0
Nasolabial angle (Col-Sn-UL) (°)	103.3	91.1	102.0 ± 8.0

FH-SN: angle between Frankfurt horizontal line and sella-nasion plane; MP-SN: angle mandibular plane (MP) and sella-nasion plane; S-Go/N-Me: the ratio of length sella-gonion to nasion-menton; SNA: sella-nasion-A point; SNB: sella-nasion-B point; ANB: A point-nasion-B point; U1-SN: upper incisor to sella-nasion plane angle; IMPA: lower incisor to MP angle; interincisal angle: angle between the mandibular and maxillary incisors; U1-NA: distance from upper incisor to NA line; L1-NA: distance from lower incisor to NA line.
